# Impaired Communication Between the Motor and Somatosensory Homunculus Is Associated With Poor Manual Dexterity in Autism Spectrum Disorder

**DOI:** 10.1016/j.biopsych.2016.06.020

**Published:** 2017-02-01

**Authors:** Abigail Thompson, Declan Murphy, Flavio Dell’Acqua, Christine Ecker, Grainne McAlonan, Henrietta Howells, Simon Baron-Cohen, Meng-Chuan Lai, Michael V. Lombardo, the MRC AIMS Consortium, and Marco Catani

**Affiliations:** a NatBrainLab, Department of Forensic, and the Sackler Institute for Translational Neurodevelopmental Sciences, Institute of Psychiatry, Psychology and Neuroscience, King’s College London; b Neurodevelopmental Sciences, and the Sackler Institute for Translational Neurodevelopmental Sciences, Institute of Psychiatry, Psychology and Neuroscience, King’s College London; c Department of Neuroimaging, and the Sackler Institute for Translational Neurodevelopmental Sciences, Institute of Psychiatry, Psychology and Neuroscience, King’s College London; d Autism Research Centre, Department of Psychiatry, University of Cambridge, Cambridge, United Kingdom; e Child and Youth Mental Health Collaborative at the Centre for Addiction and Mental Health and The Hospital for Sick Children, and Department of Psychiatry, University of Toronto, Toronto, Ontario, Canada; f Department of Psychiatry, National Taiwan University Hospital and College of Medicine, Taipei, Taiwan; g Department of Psychology and, University of Cyprus, Nicosia, Cyprus; h Center for Applied Neuroscience, University of Cyprus, Nicosia, Cyprus

**Keywords:** Autism, Diffusion tensor imaging, Homunculus, Motor skill, Primary motor cortex, Somatosensory cortex, Tractography

## Abstract

**Background:**

Fine motor skill impairments are common in autism spectrum disorder (ASD), significantly affecting quality of life. Sensory inputs reaching the primary motor cortex (M1) from the somatosensory cortex (S1) are likely involved in fine motor skill and specifically motor learning. However, the role of these connections has not been directly investigated in humans. This study aimed to investigate, for the first time, the role of the S1-M1 connections in healthy subjects in vivo and whether microstructural alterations are associated with motor impairment in ASD.

**Methods:**

Sixty right-handed neurotypical adult men aged 18 to 45 years, and 60 right-handed age- and sex-matched subjects diagnosed with ASD underwent fine motor skill assessment and scanning with diffusion tensor imaging (DTI). The streamlines of the hand region connecting S1-M1 of the motor-sensory homunculus were virtually dissected using TrackVis, and diffusion properties were extracted. The face/tongue region connections were used as control tracts.

**Results:**

The ASD group displayed lower motor performances and altered DTI measurements of the hand-region connection. Behavioral performance correlated with hand-region DTI measures in both groups, but not with the face/tongue connections, indicating anatomical specificity. There was a left-hemisphere association of motor ability in the control group and an atypical rightward shift in the ASD group.

**Conclusions:**

These findings suggest that direct interaction between S1 and M1 may contribute to the human ability to precisely interact with and manipulate the environment. Because electrophysiological evidence indicates that these connections may underpin long-term potentiation in M1, our findings may lead to novel therapeutic treatments for motor skill disorders.

The development of fine motor skills for precision grasping has been crucial to achieving greater control of the environment throughout evolution. This is particularly true for humans who have acquired the finest ability to manipulate objects for a wide range of activities that are characteristic of our species, from tool making to writing and artistic expression. Skillful hand motor ability depends on precise movement of the thumb and forefingers, which is under the direct control of the primary motor cortex (M1) (1).

The neurons of M1 are arranged according to a topographical map of the opposite body half. A distinct feature of this map consists of the disproportionate representation of neurons controlling those muscles capable of finely controlled movements, generally referred to as the motor homunculus (2). For instance, the largest areas in M1 are occupied by neurons controlling finger movements, followed by neurons for lips and tongue movement. A similar topographical organization has been described for the primary somatosensory cortex (S1) in the parietal lobe (i.e., the somatosensory homunculus). Here, areas dedicated to the representation of tactile and proprioceptive information from the fingers and oral region are larger than other body parts.

We have recently demonstrated in humans that the motor and somatosensory homunculi are directly connected through short U-shaped fibers running beneath the central sulcus (3). The pattern of distribution of these fibers follows the topographical organization of M1 and S1. That is, greater connections exist between finger regions compared with areas controlling other body parts. The existence of these connections in humans is consistent with previous reports supporting the role of somatosensory inputs in motor learning and precision grasping in animals (4, 5, 6). In monkeys, inactivation of S1 leads to altered finger coordination, such as the inability to oppose the thumb and forefinger and the inaccurate control of grip forces (4, 7). Furthermore, experimental studies in healthy humans have demonstrated that in conditions of digital anesthesia, where tactile sensation is absent, coordination of thumb and finger movements is impaired due to misalignment of fingers and an imbalance of the pressure applied (8). These studies suggest that direct connections between S1 and M1 may play a crucial role in precision grasping movements, although direct experimental evidence for this is lacking in humans (9, 10).

In the present study we therefore sought evidence of the role of S1-M1 connections in fine motor skill and precision grasping ability. To investigate this, first we combined behavioral measurements of fine motor skill performance with diffusion tensor tractography in a group of 60 healthy adults to understand the association between grasping performance and microstructural properties of U-shaped fibers connecting S1 to M1 of the hand region. As a control tract we also investigated the U-shaped connections of the face/tongue region, the microstructure of which would not be predicted to correlate with finger dexterity.

Second, we obtained diffusion tractography and grasping performance in a group of adults with a neurodevelopmental disorder in which precision grasping abnormalities are prevalent, namely autism spectrum disorder (ASD). ASD affects approximately 1% of the population and is diagnosed on the basis of social-communication impairments, alongside repetitive and stereotypic behaviors (11). Motor abnormalities have been reported in up to 79% of people with ASD (12). These abnormalities include precision grasping impairments (13). Motor impairments are present across the spectrum of autism (14) and are reported to be some of the earliest signs of ASD to emerge in infancy (15). Motor difficulties can significantly reduce day-to-day quality of life because of altered peer group interactions through sport and other social activities and increased dependence on others (16). Furthermore, motor proficiency is a necessary prerequisite for interaction with the environment, which underpins the development of social and language skills (17), highlighting the importance of investigating motor deficits in ASD. ASD is also associated with the abnormal development of white matter connections. A large number of studies have found that children and adults with ASD display structural differences in white matter tracts and across multiple brain regions (18). We therefore investigated whether abnormal structure of the S1-M1 U-shaped fibers underpins precision grasping difficulties in 60 right-handed adult men with ASD.

## Methods and Materials

### Participants

Sixty neurotypical adult men aged 18 to 45 years, and 60 age- and sex-matched subjects with a diagnosis of ASD were recruited at the Institute of Psychiatry, Psychology and Neuroscience, King’s College London, or the Autism Research Centre, University of Cambridge, as part of the UK Medical Research Council Autism Imaging Multicentre Study. Approximately equal ratios of cases to controls were recruited at each site: Institute of Psychiatry, Psychology and Neuroscience, 34:32, University of Cambridge, 26:28. All participants were right-handed, as indicated by a score of +40 or higher on the Edinburgh Handedness Inventory (19).

Exclusion criteria for all subjects included any medical illness affecting brain function or history of epilepsy, intellectual disability, major psychiatric disorder such as psychosis and attention-deficit/hyperactivity disorder (ADHD), head injury, or genetic disorder associated with autism. Participants taking any current psychotropic medications, including antipsychotic medication, mood stabilizers, benzodiazepines, stimulants, and selective serotonin reuptake inhibitors, or with a history of substance abuse were excluded. ASD participants met the ICD-10 research criteria. This was confirmed with the Autism Diagnostic Interview-Revised (20). All cases with ASD met Autism Diagnostic Interview-Revised algorithm cutoff values in the three domains of impaired reciprocal social interaction, communication, and repetitive behaviors; however, one point below cutoff in one of the domains was permitted (Table 1).

Current symptoms were assessed using the Autism Diagnostic Observation Schedule (21) but not as inclusion criteria. All participants underwent a neuropsychological test battery (22). This included the Wechsler Abbreviated Scale of Intelligence (23) as a measure of overall intellectual ability. All participants fell within the high-functioning range on the autism spectrum as defined by a full-scale IQ of >70. Written consent was acquired for all participants after a complete description of the study was given, in accordance with ethics approval by the National Research Ethics Committee, Suffolk, England.

### Motor Assessment

**Figure 1:**
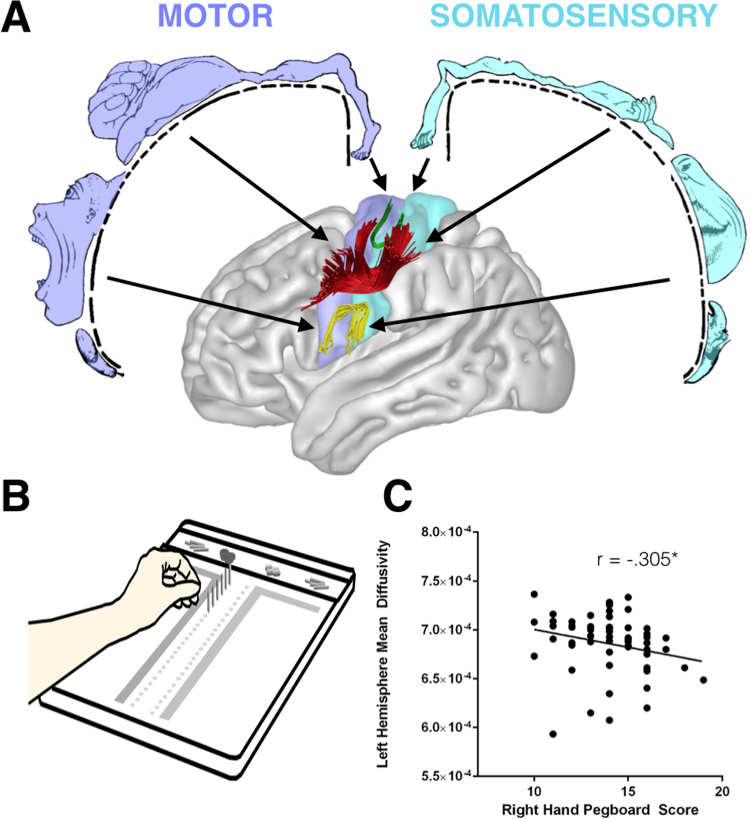
**(A)** The frontoparietal U-shaped connections of the foot, hand, and face/tongue regions, and **(B)** relation between diffusion measures of the hand-region tract and performance **(C)** in healthy controls. *Statistically significant at *p* < .025.

The Purdue Pegboard Test was selected to assess fine motor skill (24). The Purdue Pegboard is an established test of finger and hand dexterity and precision grasping ability with good test-retest reliability in both healthy subjects (25) and clinical populations (26). The participant is verbally instructed to place pins in one of two columns on a test board within a specified time period (Figure 1). There are five subtests giving five subscores. These are right hand (dominant hand), left hand (nondominant hand), both hands alternately (both hands), and a bimanual “assembly” task (Supplement). The fifth score is a composite of performance on the right hand + left hand + both hand tasks (R + L + B).

### Diffusion Tensor Imaging Data Acquisition and Preprocessing

Participants were scanned at the Centre for Neuroimaging Sciences, Institute of Psychiatry, Psychology and Neuroscience, King’s College London, and the Department of Radiology, University of Cambridge, using two identical 3T GE Signa System scanners (General Electric, Milwaukee, WI). A total of 60 contiguous slices were acquired using a sequence fully optimized for diffusion tensor imaging (DTI), providing isotropic (2.4 × 2.4 × 2.4 mm) resolution and whole head coverage. There were 32 diffusion-weighted volume directions and 6 nondiffusion weighted volumes. The diffusion weighting was equal to a *b* value of 1300 s/mm^2^. DTI processing was performed using Explore DTI (http://www.exploredti.com). The data were corrected for eddy current distortion and subject motion, and the *b* matrix was accordingly reoriented (27). The tensor model was fitted using a nonlinear least square fitting procedure (28). DTI scalar maps, including fractional anisotropy, mean diffusivity, and perpendicular diffusivity, were calculated and exported. Whole-brain tractography was performed using an Euler-like streamline propagation algorithm with a step-size of 1 mm, fractional anisotropy threshold of 0.2, and an angle threshold of 35° (29). The whole-brain tractography was imported into TrackVis for virtual dissections (30).

### Tractography and Virtual Dissections

Virtual in vivo dissections of the tracts of interest for the left and right hemispheres were performed using TrackVis. The connections were dissected in regions corresponding to the hand, face/tongue, and foot regions of the motor-sensory homunculus (Figure 1). The foot and face/tongue region connections were dissected as control tracts (Supplement).

The dissector was blinded to subject identity and diagnosis. Thirty-one data sets (25.8%) were reversed around the midline to ensure blindness to side. All dissections were completed after ensuring intrarater reliability. This was tested with the use of 10 subjects from the present study, dissected twice by the same dissector. Reliability was tested using a two-way mixed intraclass correlation coefficient (ICC) (31). For the hand and face/tongue tracts, the ICC for single measures reached >0.90 (32). We found that the foot connections consisted of only one or two individual streamlines and were not present in a number of participants. Diffusion properties for the foot streamlines did not reach >0.90 on the ICC and were therefore excluded from all further analyses.

For each tract fractional anisotropy, perpendicular diffusivity and mean diffusivity were calculated. Alterations in these measures reflect microstructural differences that may include altered axonal integrity, compactness of fiber bundles, and myelination (33). Fractional anisotropy reflects the degree of directionality of water motion within a voxel. Although highly sensitive to microstructural differences, fractional anisotropy does not provide information on the contribution of axial or perpendicular diffusion to this process. Therefore, perpendicular diffusivity, a measure of water motion perpendicular to the fiber tract, was also included. Perpendicular diffusivity may be particularly sensitive to alterations in myelination (34). Mean diffusivity was also included.

### Statistical Analysis

Statistical comparisons of the data were performed using SPSS software version 21 for PC (SPSS Inc., Chicago, IL). A Student *t* test (two-tailed) for independent samples was used to investigate differences between controls and individuals with ASD. A paired samples *t* test was used to analyze behavioral lateralization of pegboard performance. For all *t* test comparisons, Cohen’s *d* effect sizes are reported (35). For the paired samples *t* test this is corrected for dependence between means (36). To control for possible confounds, DTI tractography outcome measurements between groups were also compared using a general linear model, with age and center included as covariates. A two-tailed Pearson correlation analysis was calculated between DTI indices and pegboard measures for the control and ASD groups individually, controlling for age and center. The results of the correlation analysis were considered significant after Bonferroni correction for multiple comparisons. Because both the subscales of the pegboard and tract-based DTI indices are highly interrelated, multiple comparison correction was calculated based on the number of tracts analyzed, leaving a threshold of *p* < .025. A *z* observation analysis was used to determine differences in Pearson’s correlation coefficient 1) between hemispheres (within group), 2) between tracts (hand-region and face/tongue region tracts) (within group), and 3) between groups.

## Results

### Relation Between Manual Dexterity and Tract Properties in the Control Group

Participants showed statistically significant faster performance when executing the task with their right hand than with the left hand (*t* = 3.11, *p* = .003, *d* = 0.40). Tractography-based measurements of the hand-region U-shaped fibers in the left hemisphere correlated with performance on the pegboard test when subjects used their right or left hand (Table 2, Figure 1C). There were no significant correlations for the U-shaped fibers of the face/tongue region and pegboard performances (Supplement). In addition, no significant correlations were found between any right-hemisphere diffusion measurement and pegboard performances.

*Z* observation analysis revealed that Pearson’s correlation between pegboard performance of the right hand and mean diffusivity of the hand-region tracts in the left hemisphere were higher than the correlation between right-hand performance and right-hemisphere hand-region tracts (*z* = −1.64, *p* = .05). Correlations between the fractional anisotropy of the hand-region tracts in the left hemisphere and pegboard performance with the left hand were significantly higher than the correlations for the face/tongue tract (*z* = 2.04, *p* = .021), and the correlation between left-hand performance and the hand-region tracts in the right hemisphere (*z* = 2.27, *p* = .012). There were also significantly higher correlations between the left-hemisphere hand-region perpendicular (*z* = −2.37, *p* = .009) and mean diffusivity (*z* = −2.15, *p* = .016) and left-hand pegboard performance, compared with correlations with the hand-region tract of the right hemisphere and the left-hand pegboard performance.

### Comparison of Manual Dexterity Performance Between the Control and ASD Groups

Behavioral asymmetry in participants with ASD was lower than for controls, and differences between right- and left-hand performances were not statistically significant (*t* = 1.96, *p* = .055). Statistically significant differences in pegboard performance between the ASD and control groups were evident for a number of measurements and included lower performance of the ASD group 1) when using their right hand (*t* = 2.08, *p* = .040, *d* = 0.38); 2) on the bimanual assembly task (*t* = 3.98, *p* = .001, *d* = 0.74); and 3) on a composite score of right hand + left hand + both hands (*t* = 2.01, *p* = .047, *d* = 0.37) (Table 3). Performances with the left hand were not significantly different from those of healthy controls.

### Comparison of Tract Properties Between the Control and ASD Groups

**Figure 2:**
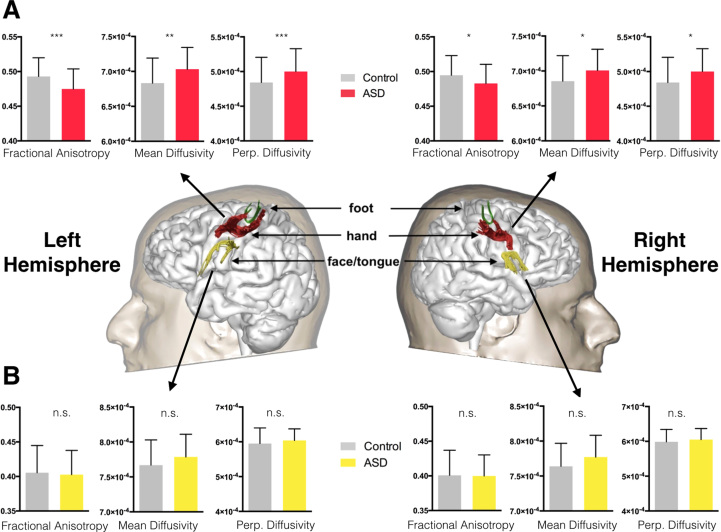
Between-group differences in fractional anisotropy, mean diffusivity, and perpendicular (Perp.) diffusivity. These were significant for **(A)** the hand-region connection but not for **(B)** the face/tongue tract. Data are mean and SD. Statistically significant at *p < .025, **p < .01, ***p < .001. ASD, autism spectrum disorder; n.s., not significant.

The tractography analysis showed that in comparison with the healthy control group, in the ASD group there was significantly decreased fractional anisotropy (*t* = 3.55, *p* = .001, *d* = 0.65) and significantly increased perpendicular diffusivity (*t* = −3.51, *p* = .001, *d* = 0.65) and mean diffusivity (*t* = −3.24, *p* = .002, *d* = 0.59) of the U-shaped fibers of the hand-region in the left hemisphere (Figure 2A). In the right hemisphere, there was significantly decreased fractional anisotropy (*t* = 2.29, *p* = .024, *d* = 0.43) and significantly increased perpendicular diffusivity (*t* = −2.48, *p* = .015, *d* = 0.46) and mean diffusivity (*t* = −2.46, *p* = .015, *d* = 0.46) of the U-shaped fibers of the hand-region; however, this did not remain significant when controlling for age and center. When controlling for age and center, only the left-hemisphere differences remained significant.

There were no significant between-group differences of the face/tongue tracts in both hemispheres (Figure 2B).

### Relation Between Manual Dexterity and Tract Properties in the ASD Group

**Figure 3:**
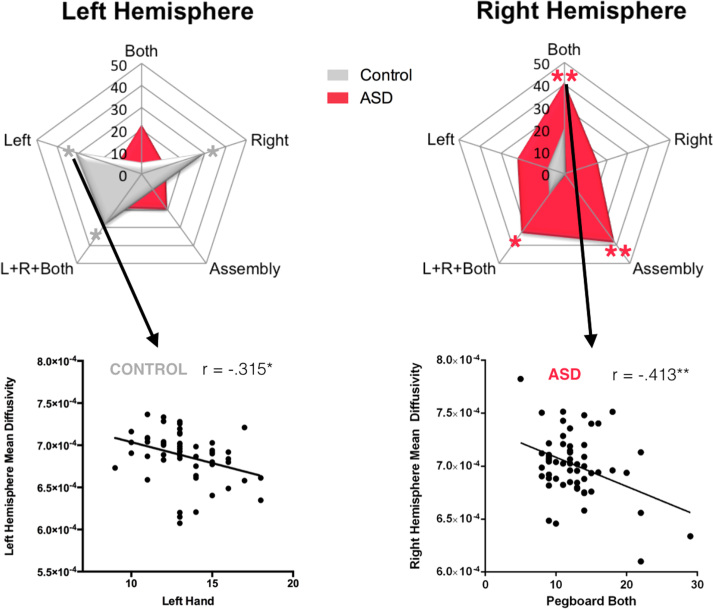
Pearson’s *r* correlations between left- and right-hemisphere hand-region tract mean diffusivity and pegboard performance in the control and autism groups. Statistically significant at *p < .025, **p < .01. ASD, autism spectrum disorder; L, left; R, right.

Unlike the healthy control group, in the ASD group there were no significant correlations between pegboard performances and diffusion measurements in the left-hemisphere hand-region tract; conversely, there were a number of significant correlations between pegboard performances and diffusion measurements in the right-hemisphere hand-region tract (Table 4, Figure 3). There were no significant correlations between manual dexterity scores and DTI measures of the face/tongue tract (Supplement).

*Z* observation analysis revealed that the correlation between pegboard performance with both hands and perpendicular diffusivity was significantly higher for the right-hemisphere hand-region tract in comparison with the right face/tongue tract (*z* = −2.29, *p* = .011). There were also significantly higher correlations between the right-hemisphere hand-region tract mean diffusivity and both pegboard performance with both hands (*z* = −2.46, *p* = .007) and performance in the composite score of R + L + B (*z* = −1.92, *p* = .027), compared with the correlations with the mean diffusivity of the face/tongue tract.

Finally, there was a significant difference in strength of correlation between the right-hemisphere hand-region tract mean diffusivity and assembly scores (*z* = −2.19, *p* = .014) when comparing the ASD and control groups.

## Discussion

The present study provides the first direct support of the role of connections between the M1 and S1 in fine motor skill performance. This finding was specific to the S1-M1 connections of the hand-region of the motor-sensory homunculus and was not present for the S1-M1 connections of the face/tongue region, demonstrating that the U-shaped white matter fibers connecting either side of the central sulcus display functional topographical organization (2). Disruption of the S1-M1 connections was associated with precision grasping impairments in a group of individuals with ASD. In comparison with healthy controls, participants with ASD showed a slower performance on the pegboard test and showed decreased fractional anisotropy and increased perpendicular diffusivity and mean diffusivity in the left hemisphere. These differences in diffusion measurements have previously been associated with alterations to tract structure, such as reduced tract coherence and organization, and reduced myelination (37) and may be associated with reduced conduction speed (38). These processes may contribute to ASD pathology (39, 40), because studies report lower myelin content in areas of the frontal lobes (41), and increased transmission times of brainstem auditory-evoked potentials in ASD (42). In addition, lower tract coherence may be linked to abnormally low signal to noise, which has been proposed to underpin ASD symptoms (43). Such pathology might reasonably be expected to degrade performance in the pegboard test in the ASD group. This is consistent with the existence of significant correlations between slower performances in the pegboard test and tractography measurements in the ASD group.

The association between the S1-M1 connections and precision grasping is asymmetrical and present only in the left hemisphere in the control group. The finding of asymmetry is in line with previous clinical studies on patients with acquired apraxia, in which a loss of grasping abilities in the left or right hand is invariably associated with left-hemisphere lesions (44, 45). Unlike the healthy control group, in the ASD group there were no significant correlations with the left-hemisphere tract, but there were a number of significant correlations with the right-hemisphere tract. Together these data suggest that the left hemisphere is dominant for precision grasping in healthy individuals and that the loss of this typical left dominance is associated with reduced fine motor skills in individuals with ASD. Loss of hemispheric dominance in the ASD group is in accordance with studies that suggest an atypical right-hemispheric shift of lateralization may be a fundamental feature of brain organization in ASD (46). Atypical rightward lateralization in ASD has most frequently been linked to language abnormality (47, 48, 49), but studies that report a rightward shift of lateralization across widespread brain areas suggest that atypical lateralization in ASD may result from a generalized maturational disturbance (46, 50).

A number of considerations must be taken into account when interpreting the present findings. Although the pegboard task measures motor speed, cognitive factors such as motivation or comprehension of task instructions may also affect the speed of pegboard completion. Although there were no significant differences in IQ between the ASD and control groups in the present study, we cannot rule out the possibility that other cognitive factors, such as attention, may have played a role in the difference in pegboard scores observed. In addition, our sample is restricted to a high-functioning ASD group, with relatively low Autism Diagnostic Observation Schedule scores. Our findings may not, therefore, be generalizable to low-functioning individuals with ASD. We also excluded participants with comorbid ADHD. Although this may be considered a strength of the sample, it also limits the generalizability of our findings to individuals with ASD and comorbid ADHD. ADHD may be present in up to 78% of individuals with ASD (51, 52), and studies suggest the nature of motor impairments may be distinct in individuals with ASD with and without ADHD (53). Future studies should therefore aim to investigate this.

Furthermore, the motor impairments we report in the ASD group may relate to abnormalities in other brain regions, in addition to the S1-M1 connections. Cerebellar (54) and thalamic (55) abnormalities have been reported in ASD, including altered white matter connectivity of these regions (56, 57). Abnormality of these regions may have a distinct influence on motor impairment in ASD. For example, although the S1-M1 connections likely play a role in sensory feedback after contact with an object, anterior cerebellar abnormalities may be associated with deficits in the feedforward planning, which occurs before object contact (58). A more comprehensive assessment of the tracts involved in motor planning and execution, which may also include, for example, the superior cerebellar peduncle and the superior longitudinal fasciculus system, will be necessary to understand the specific role of each tract to fine motor skills in ASD. Because of the characteristics of our data set, we were unable to perform dissections of the superior longitudinal fasciculus for which high angular resolution diffusion-weighted imaging models that necessitate higher number of directions and higher *b* values are required (59).

Several theories of disconnection in ASD propose a dual mechanism of increased connectivity in short-range connections and reduced connectivity in long-range connections (43). The present study reports alterations in U-shaped fibers between frontal and parietal lobes, which are anatomically considered to be short tracts compared with other association pathways. However, the concept of short- and long-range connectivity in ASD is not well defined (51), and indeed other studies have previously reported reductions in short-range white matter connectivity in ASD (52, 53). Future studies will be required to determine whether the short- versus long-range dichotomy defined at the anatomical and functional levels accurately reflects underlying biology in ASD. Such studies will require advanced methods to quantify connectivity of intracortical fibers, U-shaped fibers, and long interlobar fibers.

The S1-M1 connections are thought to be the terminal component of an indirect route for thalamic sensory information to reach M1, as opposed to a direct thalamo-M1 pathway (3). Electrophysiological studies in cats indicate that this indirect route may play a role in motor learning. For example, tetanic stimulation of S1 leads to long-term potentiation (LTP) in M1 (5), an effect that does not occur with tetanic stimulation of the thalamus alone (6). Further evidence in support of the role of the S1-M1 connections in motor learning comes from the finding that ablation of S1 in macaque monkeys prevents the ability to learn novel motor sequences (60). Our study is restricted to an adult cohort, and it is not possible to establish whether the microstructural abnormalities reported are due to processes occurring early or late in development. However, white matter differences have been reported across the lifespan in ASD, including early childhood (61). Because of the involvement of the S1-M1 connections in LTP and motor learning, as suggested by the above studies in nonhuman animal models, the findings of the present study may lead to novel therapeutic approaches targeting motor learning in young children with ASD and in children with developmental dyspraxia in general. Navigated transcranial magnetic stimulation of the S1 cortex, for example, could be used to elicit LTP in M1 and to facilitate consolidation of behaviorally induced motor learning. Because motor disturbance is one of the earliest signs of abnormality in infants with ASD and underpins later abnormal development of language and social abilities (15, 17), the development of a therapeutic approach for motor impairments in ASD would be of great importance.

In conclusion, we reported significant correlations between pegboard performance skill and the microstructural properties of white matter connections between S1 and M1 of the left-hand region in a group of healthy adults. We also found that poor pegboard performance was associated with structural abnormality of this tract in a clinical population of individuals with ASD. Our findings represent the first empirical evidence in humans that development of normal S1-M1 connections play an important role in fine motor control. In addition, because S1 input to M1 underpins LTP in M1, these findings may lead to novel therapeutic approaches for motor rehabilitation in people affected by ASD, and in individuals with specific motor learning disability.

## Figures and Tables

**Table 1:** Subject Demographic Characteristics

Characteristic	Healthy Controls	Subjects With Autism
	(*n* = 60)	(*n* = 60)
Age, Years^a^	29 (7) [18–45]	26 (7) [18–43]
WASI IQ Score^a^		
Full scale	111 (12) [88–133]	115 (12) [77–137]
Verbal	108 (13) [84–139]	112 (13) [71–137]
Performance	111 (13) [88–133]	115 (13) [75–137]
ADI-R Score		
Total	NA	39 (10) [21–62]
Social	NA	18 (5) [9–28]
Communication	NA	14 (4) [8–24]
Repetitive	NA	5 (2) [2–10]
ADOS Score^b^		
Total	NA	11 (5) [1–23]
Social	NA	6 (3) [1–14]
Communication	NA	3 (2) [0–7]
Repetitive	NA	1 (1) [0–6]

Data are mean (SD) [range].

ADI-R, Autism Diagnostic Interview-Revised; ADOS, Autism Diagnostic Observation Schedule; NA, not applicable; WASI, Wechsler Abbreviated Scale of Intelligence.

a No significant between-group differences were found in age, full-scale IQ, verbal IQ, or performance IQ (all *p* > .05, 2-tailed).

b Information was available for 58 subjects with autism.

**Table 2:** Correlations Between Pegboard Performance and Hand-Region Tract-Specific Measurements for the Control Group (Controlling for Age and Center)

Pegboard	Diffusion Tensor Measures of Hand-Region Frontoparietal U Tract					
	Left Hemisphere			Right Hemisphere		
	Fractional	Perpendicular	Mean	Fractional	Perpendicular	Mean
	Anisotropy	Diffusivity	Diffusivity	Anisotropy	Diffusivity	Diffusivity
Right	.074	−.213	−.305^a^	−.140	.030	−.008
Left	.352^b^	−.376^b^	−.315^a^	−.057	.049	.077
Both	.199	−.157	−.051	.169	−.192	−.212
R + L + Both	.244	−.316^a^	−.287^a^	.021	−.095	−.116
Assembly	.183	−.157	−.090	.002	.005	.009

Values are Pearson’s *r*.

R + L + Both, right hand + left hand + both composite score.

a *p* < .025.

b *p* < .01.

**Table 3:** Comparison of Purdue Pegboard Test Scores Between Control and Autism Groups

Purdue Pegboard Test	Control	Autism	*t*
Right	14 (2)	13.1 (2.4)	2.08^a^
Left	13.3 (2)	12.6 (2.8)	1.57
Both	13.6 (2.9)	12.6 (4.3)	1.39
R + L + Both	40.7 (4.8)	38.3 (7.8)	2.01^a^
Assembly	34.6 (8.2)	28.3 (8.9)	3.97^b^

Values are mean (SD).

L, left hand; R, right hand.

a *p* < .05.

b *p* < .001.

**Table 4:** Correlations Between Pegboard Performance and Hand-Region Tract-Specific Measurements for the ASD Group (Controlling for Age and Center)

Pegboard	Diffusion Tensor Measures of Hand-Region Frontoparietal U Tract					
	Left Hemisphere			Right Hemisphere		
	Fractional	Perpendicular	Mean	Fractional	Perpendicular	Mean
	Anisotropy	Diffusivity	Diffusivity	Anisotropy	Diffusivity	Diffusivity
Right	−.029	−.053	−.112	.080	−.175	−.158
Left	.175	−.135	−.121	.116	−.214	−.221
Both	.256	−.253	−.223	.280^a^	−.413^b^	−.413^b^
R + L + Both	.176	−.186	−.187	.201	−.331^a^	−.328^a^
Assembly	.136	−.182	−.196	.050	−.289	−.382^b^

Values are Pearson’s *r*.

R + L + Both, right hand + left hand + both composite score.

a *p* < .025.

b *p* < .01.
