# Supplementary material for: Impaired Communication Between the Motor and Somatosensory Homunculus Is Associated With Poor Manual Dexterity in Autism Spectrum Disorder
Source: Biol Psychiatry. 2017 Feb 1;81(3):211–9. doi: 10.1016/j.biopsych.2016.06.020 (PMC5227100; doi:10.1016/j.biopsych.2016.06.020)
Supplement: Supplementary file 1 — Supplementary material [file mmc1.pdf]

Impaired Communication Between the Motor and Somatosensory Homunculus Is Associated With Poor Manual Dexterity in Autism Spectrum Disorder

Supplemental Information

Supplementary Methods & Materials

Motor Assessment

The pegboard test includes a test board that is 12 x 18 inches in size and has two columns in the middle, each of which has 25 holes. There are pins, collars and washers located in two cups at the top of the board. The participant is verbally instructed to place pins in one of the two columns within a specified time period (Figure 1). For each of the subtests, the score is the total number of pins placed. There are four subtests giving five subscores: right hand (dominant hand), left hand (non-dominant hand), both hands alternatively and assembly. In the right hand and left hand subtests the participant is required to place as many pegs as possible into the right or left row with their right and left hands respectively, within a time limit of 30 seconds. The both hands subtest requires the participant to use both hands alternatively to place as many pins as possible in both rows in 30 seconds. The assembly subtest requires the participant to use both hands consecutively, first placing a pin in the board (right hand), then placing a washer on top of the pin (left hand), then placing a collar on top (right hand) and finally another collar on top (left hand). This procedure has to be completed before the participant can move on to the next pin, completing as many as possible in a time limit of 60 seconds. The fifth score is a composite score of performance on the right + left hand + both hand (R + L + B) tasks.

Description of Virtual Dissections

Based on previous tractography work (1), regions of interest were defined manually on the axial, coronal and sagittal fractional anisotropy images of each participant, and were used as

seed regions for tracking. All white matter connections projecting between S1 and M1 were captured using a two-region of interest (ROI) approach. The central sulcus was isolated on the axial plane, using the inverted omega as the radiological guide. A first region of interest was manually traced along the extent of the precentral gyrus (corresponding to M1) in a dorsal-to-ventral manner, to incorporate the body of white matter within the gyrus. A second region of interest was traced along the extent of the postcentral gyrus (corresponding to S1) in a dorsal-to-ventral manner. All white matter connections projecting between the two ROIs were identified. The connections of the hand region were identified using the anatomical landmark of the inverted omega of the precentral gyrus. All connections between M1 and S1 surrounding this anatomical landmark were mapped using new regions of interest, to isolate the region corresponding to the motor and somatosensory maps for the hand (2). The foot and face/tongue tracts were identified as the connections between M1 and S1 located along the most dorsal, and ventral aspect of the central sulcus, respectively (separate to the hand region tract). These connections were isolated using new regions of interest. For all tracts, extraneous or artefactual streamlines were excluded using a not-ROI.

Correlations

Table S1. Correlations between pegboard performance and face/tongue-region tract-specific measurements for the control group (controlling for age and center).

	Diffusion Tensor Measures of Hand-Region Fronto-Parietal U Tract					
	Left Hemisphere			Right Hemisphere		
	Fractional Anisotropy	Perpendicular Diffusivity	Mean Diffusivity	Fractional Anisotropy	Perpendicular Diffusivity	Mean Diffusivity
Pegboard						
Right	.040	-.216	-.241	-.241	-.126	-.189
Left	-.060	-.106	-.158	-.191	-.236	-.305
Both	.190	-.168	-.112	-.086	-.094	-.086
R+L+Both	.047	-.205	-.240	-.229	-.203	-.229
Assembly	-.137	.163	.181	-.045	-.015	-.045

Values are Pearson’s r. Significance is * $p < 0.025$, ** $p < 0.01$, *** $p < 0.001$.
R+L+Both: Right hand + left hand + both composite score.

Table S2. Correlations between Pegboard performance and face/tongue-region tract-specific measurements for the ASD group (controlling for age and center).

	Diffusion Tensor Measures of Hand-Region Fronto-Parietal U Tract					
	Left Hemisphere			Right Hemisphere		
	Fractional Anisotropy	Perpendicular Diffusivity	Mean Diffusivity	Fractional Anisotropy	Perpendicular Diffusivity	Mean Diffusivity
Pegboard						
Right	.154	-.104	-.057	.227	-.036	.084
Left	.201	.072	.020	.296	-.134	.037
Both	-.109	.196	.141	.075	.004	.052
R+L+Both	.072	.034	.057	.225	-.059	.069
Assembly	.053	-.027	-.005	.131	-.054	-.004

Values are Pearson’s r. Significance is * $p < 0.025$, ** $p < 0.01$, *** $p < 0.001$.
R+L+Both: Right hand + left hand + both composite score.

Supplemental References

1. Catani M, Dell'acqua F, Vergani F, Malik F, Hodge H, Roy P, et al. (2012): Short frontal lobe connections of the human brain. *Cortex*. 48:273-291.
2. Penfield W, Boldrey E (1937): Somatic motor and sensory representation in the cerebral cortex of man as studied by electrical stimulation. Brain: A journal of neurology., pp 389-443.
